# Breast Cancer Stem Cell Potency of Nickel(II)‐Polypyridyl Complexes Containing Non‐steroidal Anti‐inflammatory Drugs

**DOI:** 10.1002/chem.202001578

**Published:** 2020-09-30

**Authors:** Catherine J. Feld, Alice Johnson, Zhiyin Xiao, Kogularamanan Suntharalingam

**Affiliations:** ^1^ School of Chemistry University of Leicester Leicester LE1 7RH UK

**Keywords:** antitumor agents, bioinorganic chemistry, cyclooxygenase-2, necroptosis, nickel

## Abstract

We report the breast cancer stem cell (CSC) potency of two nickel(II)‐3,4,7,8‐tetramethyl‐1,10‐phenanthroline complexes, **1** and **3**, containing the non‐steroidal anti‐inflammatory drugs (NSAIDs), naproxen and indomethacin, respectively. The nickel(II) complexes, **1** and **3** kill breast CSCs and bulk breast cancer cells in the micromolar range. Notably, **1** and **3** display comparable or better potency towards breast CSCs than salinomycin, an established CSC‐active agent. The complexes, **1** and **3** also display significantly lower toxicity towards non‐cancerous epithelial breast cells than breast CSCs or bulk breast cancer cells (up to 4.6‐fold). Mechanistic studies suggest that **1** and **3** downregulate cyclooxygenase‐2 (COX‐2) in breast CSCs and kill breast CSCs in a COX‐2 dependent manner. Furthermore, the potency of **1** and **3** towards breast CSCs decreased upon co‐treatment with necroptosis inhibitors (necrostatin‐1 and dabrafenib), implying that **1** and **3** induce necroptosis, an ordered form of necrosis, in breast CSCs. As apoptosis resistance is a hallmark of CSCs, compounds like **1** and **3**, which potentially provide access to alternative (non‐apoptotic) cell death pathways could hold the key to overcoming hard‐to‐kill CSCs. To the best of our knowledge, **1** and **3** are the first compounds to be associated to COX‐2 inhibition and necroptosis induction in CSCs.

## Graphical abstract

**Kill switch**: Ni^II^‐polypyridyl complexes bearing non‐steroidal anti‐inflammatory drugs are shown to potently kill breast cancer stem cells (CSCs) via a cyclooxygenase‐2 (COX‐2)‐dependent pathway and in a manner that is curtailed by necroptosis inhibitors. As COX‐2 overexpression and apoptosis dysfunction are inherent to breast CSCs, the compounds presented here could help in the fight against hard‐to‐kill breast CSCs.

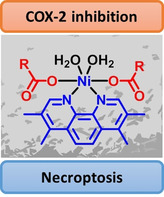

## Introduction

Cancer stem cells (CSCs) are a subpopulation of solid and blood tumours with self‐renewal properties.^[1]^ Their relatively slow cell proliferation rates allow them to evade most therapeutic regimens such as chemotherapy and radiation, which target fast growing bulk cancer cells.^[2]^ The very low proportion of CSCs within a given tumour (sometimes <1 % of the population) and their tendency to reside in hard to reach niches, means they are often missed by surgery as well.^[3]^ Therapy‐resistant CSCs have the potential to reform tumour mass within the primary site or promote cancer cell motility and tumour anchorage at secondary sites.^[4]^ Therefore, CSCs are widely thought to contribute to metastasis and relapse.[^1a^, ^5^] Given our understanding of tumour heterogeneity and CSCs, effective therapeutic intervention in cancer patients must involve the removal of all types of cancer cells, including CSCs. A number of CSC characteristics have been identified such as cell surface markers, deregulated signalling pathways, and components within the microenvironments in which they reside, however, despite the best efforts of academic‐ and pharmaceutical‐driven approaches and several on‐going and planned clinical trials, there are still no clinically approved agents that can remove CSCs at their therapeutically administered dose.^[6]^ The vast majority of chemical agents investigated as potential anti‐CSC agents are completely organic in nature.^[6a]^ We and others have recently shown that the chemical and physical diversity offered by metals can be harnessed to develop inorganic compounds with promising anti‐CSC activities.^[7]^

We recently developed a number of copper(II)‐phenanthroline complexes containing nonsteroidal anti‐inflammatory drugs (NSAIDs) capable of killing breast CSCs and bulk breast cancer cells in vitro.^[8]^ Mechanistic studies showed that the copper(II) complexes induce breast CSC death by elevating reactive oxygen species (ROS) levels and inhibiting cyclooxygenase‐2 (COX‐2). We attributed the success of this approach to the vulnerability of breast CSCs and bulk breast cancer cells to changes in their intracellular redox state and the overexpression of COX‐2 in breast CSCs and bulk breast cancer cells.^[9]^ We also recently reported a nickel(II)‐phenanthroline‐dithiocarbamate complex capable of killing breast CSCs in the micromolar range.^[10]^ Mechanistic studies revealed that the nickel(II) complex displayed all the hallmarks of necroptosis such as necrosome‐mediated cell membrane disruption and mitochondrial depolarisation, and distinctive necroptotic morphological features. Furthermore, unbiased predictive functional genetic analysis based on RNA interference (RNAi) proved that the mechanism of action of the nickel(II) complex resembled that of shikonin, a *bona fide* necroptosis inducer.^[11]^ Given that apoptosis resistance is a well‐established characteristic of therapy‐resistant CSCs,^[1b]^ compounds which can evoke cell death through non‐apoptotic pathways, such as necroptosis, could help overcome apoptosis‐resistant CSCs. Despite the very promising anti‐breast CSC activities of the copper(II)‐phenanthroline‐NSAID complexes and the nickel(II)‐phenanthroline‐dithiocarbamate complex reported by us thus far, potential in vivo application and further preclinical development is limited by their relative instability in biologically relevant solutions.[^8b^, ^10^] Here, we have sought to combine the beneficial anti‐CSC properties of the copper(II)‐phenanthroline‐NSAID complexes (COX‐2 inhibition) and the nickel(II)‐phenanthroline‐dithiocarbamate complex (necroptosis‐inducing properties) and improve their stability in biologically relevant solutions, by developing nickel(II)‐phenanthroline complexes containing naproxen and indomethacin (potent COX‐2 inhibitors).

## Results and Discussion

### Synthesis, characterisation, and stability studies

**Figure 1:**
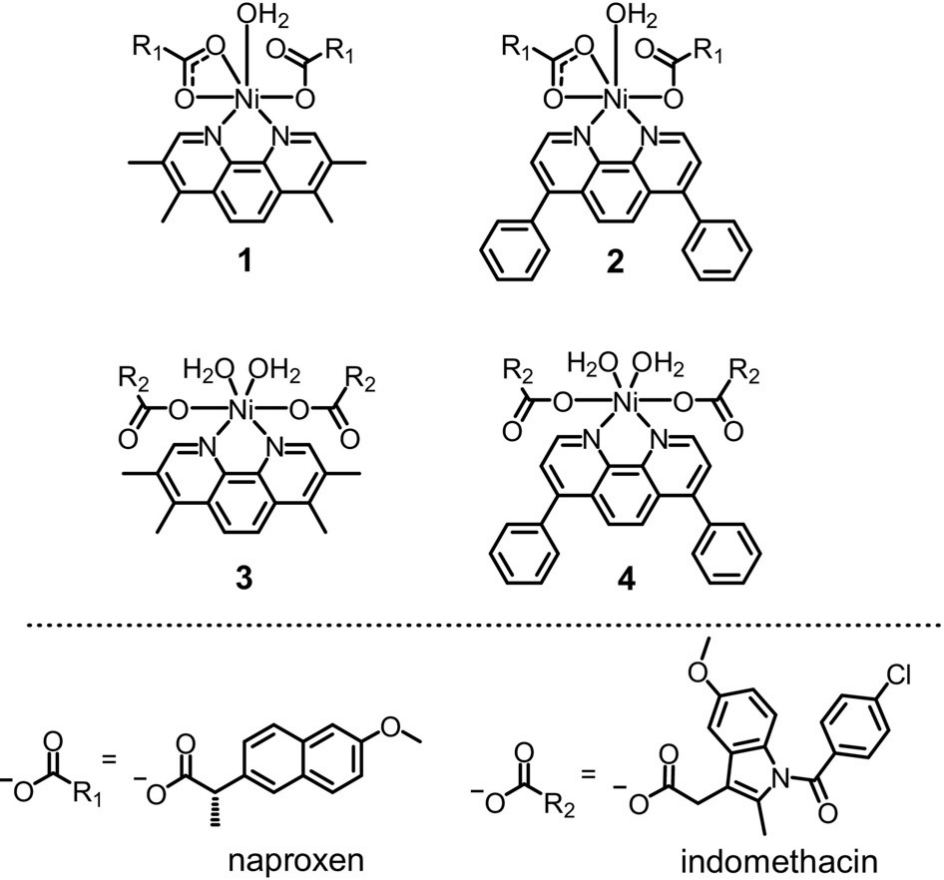
Chemical structures of the nickel(II)‐nonsteroidal anti‐Inflammatory drug complexes, **1**–**4** under investigation in this study.

The nickel(II)‐phenanthroline‐NSAID complexes, **1**–**4** synthesised in this study are shown in Figure 1. The nickel(II) complexes, **1**–**4** were prepared by reacting NiCl_2_⋅6 H_2_O with 3,4,7,8‐tetramethyl‐1,10‐phenanthroline or 4,7‐diphenyl‐1,10‐phenanthroline and two equivalence of naproxen or indomethacin in methanol, under basic conditions. The nickel(II) complexes, **1**–**4** were isolated as pale green or green solids in good yields (60–90 %) and fully characterized by infra‐red and UV/Vis spectroscopy, and elemental analysis (Figures S1, S2). The difference (Δ) between the vibrational stretching frequencies between the asymmetric, *ν*_asym_(CO_2_) and symmetric, *ν*_sym_(CO_2_) carbonyl peaks gives an indication into the binding mode of the associated carboxylic acid group to a given metal centre.^[12]^ According to the ATR‐FTIR spectra of **1**–**4**, the difference (Δ) between the *ν*_asym_(CO_2_) and *ν*_sym_(CO_2_) stretching bands for **1** and **2** varied between 213–214 cm^−1^ (Figures S1A, B), indicative of a mixed monodentate‐bidentate binding mode for the carboxylate group on naproxen to the nickel(II) centre (as depicted in Figure 1). The Δ(CO_2_) stretching bands for **3** and **4** varied between 235–238 cm^−1^ (Figures S1C, D), indicative of a monodentate binding mode for the carboxylate group on indomethacin to the nickel(II) centre (as depicted in Figure 1). The carboxylate group binding mode assignments for **1**–**4** are fully consistent with previous reports on structurally similar nickel(II)‐phenanthroline complexes bearing naproxen and indomethacin.^[13]^ The UV/Vis spectra of **1**–**4** (50 μm) displayed intense bands between 275–282 nm which are tentatively assigned to π–π* and metal‐perturbed π–π* transitions involving both the corresponding phenanthroline and NSAID ligands (Figure S2). Weaker bands around 304–320 nm and 329–358 nm are tentatively assigned to high energy metal‐to‐ligand charge‐transfer (MLCT) and typical MLCT (d‐π*) transitions (Figure S2). The purity and composition of **1**–**4** was confirmed by elemental analysis (see Experimental Section).

The lipophilicity of the nickel(II) complexes, **1**–**4** was determined by measuring the extent to which they partitioned between octanol and water, P. The experimentally determined LogP values for **1**–**4** varied between 0.63±0.05 and 1.52±0.14 (Table S1). The LogP values for **1**–**4** are consistent with those reported for related copper(II)‐ and manganese(II)‐phenanthroline‐NSAID complexes.[^8b^, ^14^] The hydrophobic nature of **1**–**4** suggests that they will be readily taken up by cells. UV/Vis spectroscopy studies were carried out to assess the stability of **1**–**4** in biological relevant solutions. The UV/Vis π–π* and MLCT absorption bands of the 3,4,7,8‐tetramethyl‐1,10‐phenanthroline‐bearing complexes, **1** and **3** (50 μm) in PBS:DMSO (200:1) remained constant over the course of 24 h at 37 °C suggestive of stability (Figures S3, S4). In contrast, the 4,7‐diphenyl‐1,10‐phenanthroline‐containing complexes, **2** and **4** (50 μm) were relatively unstable in PBS:DMSO (200:1) (Figures S5, S6). The UV/Vis bands associated to **1** and **3** (50 μm) in mammary epithelial cell growth medium (MEGM)/DMSO (200:1) remained unaltered over the course of 24 h at 37 °C, suggestive of stability in conditions required for cell‐based studies (Figures S7, S8). Under the same conditions, **2** and **4** were relatively unstable (Figures S9, S10). Collectively, the UV/Vis spectroscopy studies suggest that the stability of the complexes in biologically relevant solutions is highly dependent on the polypyridyl ligand, with the nickel(II)‐NSAID complexes containing 3,4,7,8‐tetramethyl‐1,10‐phenanthroline (**1** and **3**) being more stable than those with 4,7‐diphenyl‐1,10‐phenanthroline (**2** and **4**).

### Breast cancer stem cell, bulk breast cancer, and normal breast cell potency in monolayer systems

Given the instability of the 4,7‐diphenyl‐1,10‐phenanthroline‐containing complexes, **2** and **4** in MEGM (Figures S9, S10), cellular studies were not performed with these complexes. The cytotoxicity of **1** and **3** against bulk breast cancer cells (HMLER) and breast CSC‐enriched cells (HMLER‐shEcad) was determined using the MTT assay. The IC_50_ values were determined from dose‐response curves (Figures S11, S12) and are summarised in Table 1. The nickel(II) complexes, **1** and **3** exhibited micromolar potency towards both HMLER and HMLER‐shEcad cells, comparable to salinomycin (an established breast CSC‐active agent).^[15]^ Notably, **1** and **3** displayed higher potency (*p*<0.05, *n*=12) for CSC‐enriched HMLER‐shEcad cells than CSC‐depleted HMLER cells. Although the corresponding copper(II) complex with naproxen, **Cu‐1** (see Figure S13 for chemical structure) displayed slightly better selectivity for CSCs over bulk cancer cells than **1** and **3**,^[8b]^ both **1** and **3** exhibited a larger toxicity differential (the concentration difference between the IC_50_ values for HMLER and HMLER‐shEcad cells). The toxicity differential for **Cu‐1** is 0.26 μm whereas the differential for **1** and **3** is 4.69 μm and 0.91 μm, respectively, therefore **1** and **3** theoretically have a larger concentration window to treat CSCs over bulk cancer cells. The corresponding copper(II) complex with indomethacin, **Cu‐3** (see Figure S13 for chemical structure) preferentially killed HMLER cells over HMLER‐shEcad cells.^[8b]^ Strikingly, the indomethacin‐containing nickel(II) complex, **3** displayed 2.3‐fold (*p*<0.05, *n*=12) and 3.1‐fold (*p*<0.05, *n*=12) greater potency for CSC‐enriched HMLER‐shEcad cells than salinomycin and cisplatin (a platinum‐based anticancer agent), respectively.[^8a^, ^16^] The NSAIDs components, naproxen and indomethacin were previously shown to be non‐toxic towards both HMLER and HMLER‐shEcad cells (IC_50_ values >100 μm) under identical conditions.[^8a^, ^8b^] Further control cytotoxicity studies showed that NiCl_2_⋅6 H_2_O was non‐toxic towards HMLER and HMLER‐shEcad cells (IC_50_>100 μm) (Table 1 and Figure S14). Collectively, this suggests that the cytotoxicity of **1** and **3** towards breast CSCs and bulk breast cancer cells is likely to result from the intact cellular entry of the nickel(II) complexes, which allows for the synergistic co‐delivery of all the complex components.

**Table 1:** IC_50_ values of the nickel(II) complexes, **1** and **3**, the copper(II) complexes, **Cu‐1** and **Cu‐3**, cisplatin, salinomycin, and NiCl_2_⋅6 H_2_O against HMLER cells, HMLER‐shEcad cells, and HMLER‐shEcad mammospheres.

Compound	HMLER IC_50_ [μm]^[a]^	HMLER‐shEcad IC_50_ [μm]^[a]^	Mammosphere IC_50_ [μm]^[b]^
**1**	12.33±0.32	7.64±0.05	46.15±12.37
**3**	2.74±0.06	1.83±0.11	55.40±0.42
NiCl_2_⋅6 H_2_O	>100	>100	>100
**Cu‐1** ^[c]^	0.54±0.27	0.28±0.03	0.79±0.39
**Cu‐3** ^[c]^	0.59±0.25	0.79±0.06	n.d.
cisplatin^[c]^	2.57±0.02	5.65±0.30	13.50±2.34
salinomycin^[c]^	11.43±0.42	4.23±0.35	18.50±1.50

[a] Determined after 72 h incubation (mean of three independent experiments ±SD). [b] Determined after 5 days incubation (mean of three independent experiments ±SD). [c] Reported in references [8a, 8b, 16, 18]; n.d.=not determined.

To gauge therapeutic potential, the cytotoxicity of **1** and **3** towards non‐cancerous epithelial breast MCF10A cells was determined. The complexes, **1** and **3** were significantly less potent towards MCF10A cells than HMLER and HMLER‐shEcad cells (IC_50_ value for **1**=18.66(±1.96) μm, up to 2.4‐fold, *p*<0.05 and IC_50_ value for **3**=8.40(±0.52) μm, up to 4.6‐fold, *p*<0.05) (Figure S15). Therefore, according to the cytotoxicity studies in monolayer systems, **1** and **3** have the potential to preferentially kill breast CSCs and bulk breast cancer cells over non‐cancerous breast cells.

### Mammosphere inhibitory and viability studies

**Figure 2:**
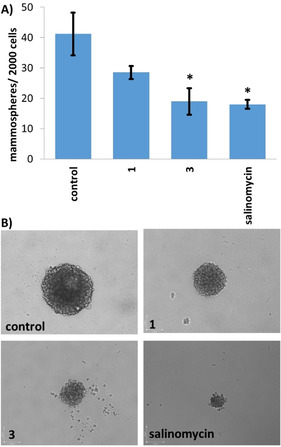
A) Quantification of mammosphere formation with HMLER‐shEcad cells untreated and treated with **1**, **3**, or salinomycin at their respective IC_20_ values for 5 days. Error bars=SD and Student t‐test, *=*p*<0.05. B) Representative bright‐field images (×10) of the mammospheres in the absence and presence of **1**, **3**, or salinomycin at their respective IC_20_ values.

Breast CSCs are able to form tumour‐like, spherical shaped structures called mammospheres when grown under serum‐free, low attachment conditions.^[17]^ The ability of the nickel(II) complexes, **1** and **3** to inhibit mammosphere formation was probed using an inverted microscope. Addition of **3** (IC_20_ value for 5 days) significantly (*p*<0.05) reduced the number and size of mammospheres formed relative to the untreated control (Figures 2 A, B). Treatment with **1** (IC_20_ value for 5 days) did not significantly (*p*>0.05) change the number of mammospheres formed, however, the nickel(II) complex did markedly reduce the size of the mammospheres formed (Figures 2 A, B). The most effective complex, **3** reduced mammosphere formation (54 %) to a comparable level as salinomycin (56 %) (Figures 2 A, B). Treatment with NiCl_2_⋅6 H_2_O (2 μm for 5 days) did not dramatically affect the number or size of mammospheres formed (Figures S16, S17). We have previously shown that indomethacin has minimal mammosphere inhibitory effects (under identical conditions).^[8a]^ Taken together, this suggests that the mammosphere inhibitory effect of **3** is likely to result from the intact delivery of the nickel(II) complex. To determine the effect of **1** and **3** on mammosphere viability, the colorimetric resazurin‐based reagent, TOX8 was used. The IC_50_ values (concentration required to reduce mammosphere viability by 50 %) of **1** and **3** were in the micromolar range (Figure S18 and Table 1). Notably, the mammosphere potency of **1** and **3** was much lower than salinomycin, cisplatin, and the corresponding copper(II) complex with naproxen, **Cu‐1** under identical conditions (Table 1).[^8b^, ^16^, ^18^] Previous reports and control experiments conducted in this study showed that NiCl_2_⋅6 H_2_O, naproxen, and indomethacin were all non‐toxic towards mammospheres (IC_50_>133 μm, Figure S18 and Table 1).^[8a]^ Therefore the mammosphere potency observed for **1** and **3** is likely to be due to the intact mammosphere uptake of the nickel(II) complexes, which facilitates the concerted co‐delivery of all the complex components.

### Mechanism of action: cellular uptake, cyclooxygenase‐2 inhibition and necroptosis induction

**Figure 3:**
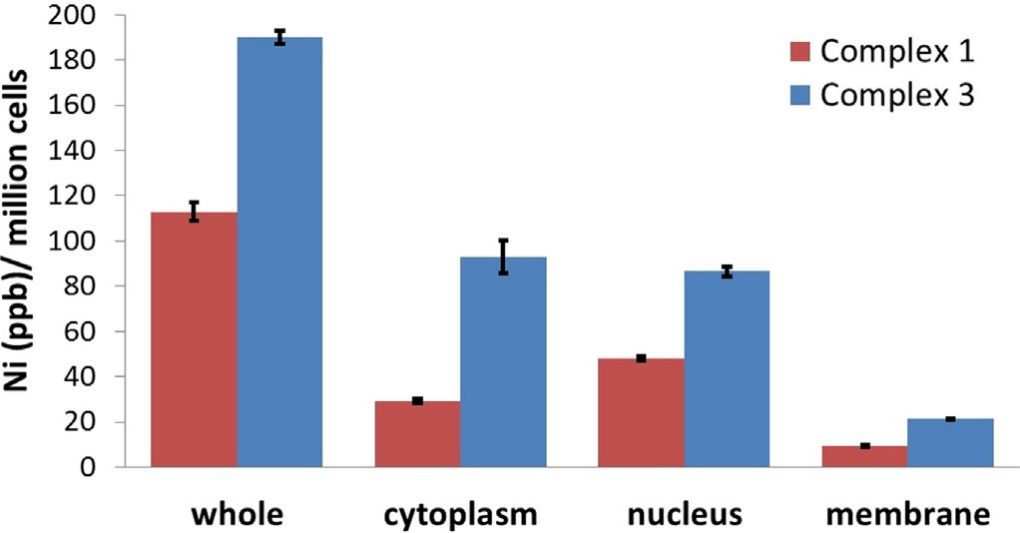
Nickel content in whole cell, cytoplasm, nucleus, and membrane fractions isolated from HMLER‐shEcad cells treated with **1** or **3** (5 μm for 24 h).

Cellular uptake studies were carried out to determine the CSC permeability of **1** and **3**. HMLER‐shEcad cells were incubated with **1** and **3** (5 μm for 24 h) and the intracellular nickel content was determined by inductively coupled plasma mass spectrometry (ICP‐MS). The complexes, **1** and **3** were readily internalised by HMLER‐shEcad cells, with 112.9(±4.2) ppb of Ni/ million cells detected for **1**‐treated cells and 190.0(±2.8) ppb of Ni per million cells detected for **3**‐treated cells (Figure 3). A clear correlation was observed between lipophilicity (LogP), cytotoxicity, and whole cell uptake. The indomethacin‐bearing complex, **3**, with a LogP value of 0.96±0.13 and an IC_50_ value of 1.83(±0.11) μm towards HMLER‐shEcad cells was internalized to a greater extent than the naproxen‐containing complex, **1** with a LogP value of 0.63±0.05 and an IC_50_ value of 7.64(±0.05) μm towards HMLER‐shEcad cells. Fractionation studies were carried out with **1**‐ and **3**‐treated HMLER‐shEcad cells (5 μm for 24 h) to determine the CSC localisation of **1** and **3** (Figure 3). A significant amount of internalised **1** and **3** was detected in the cytoplasm (26 and 49 %) and nucleus (43 and 46 %). This is consistent with the presence of the naproxen and indomethacin moieties in **1** and **3**, which target COX‐2 localised on luminal surfaces of the endoplasmic reticulum and the inner membrane of the nuclear envelope.^[19]^ Relatively lower, but appreciable, amounts of **1** and **3** were trapped in the membrane (8 and 11 %, respectively). Overall, the fractionation studies suggest that **1**‐ and **3**‐induced CSC toxicity is more likely to result from deleterious action within the cytoplasm and nucleus rather than the membrane.

**Figure 4:**
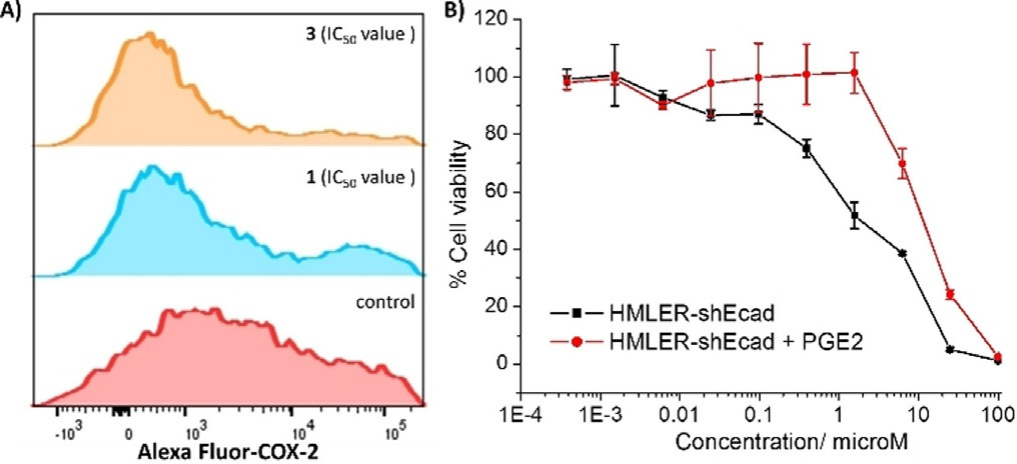
A) Representative histograms displaying the green fluorescence emitted by anti‐COX‐2 Alexa Fluor 488 nm antibody‐stained HMLER‐shEcad cells treated with LPS (2.5 μm) for 24 h (red) followed by 72 h in media containing **1** (IC_50_ value, blue) or **3** (IC_50_ value, orange). B) Representative dose‐response curves for the treatment of HMLER‐shEcad cells with **3** after 72 h incubation in the presence and absence of PGE2 (20 μm).

Breast carcinomas aberrantly express COX‐2.^[20]^ COX‐2 is heavily associated to poor prognostic markers such as large tumour size and high tumour grade.^[21]^ COX‐2 overexpression is also linked to angiogenesis, metastasis, epithelial‐to‐mesenchymal transition (EMT), and CSC maintenance and regulation.^[22]^ Remarkably, breast cancer patients who take COX‐2 inhibitors display a survival advantage.^[23]^ Given the presence of naproxen and indomethacin in **1** and **3** respectively, we investigated whether the mechanism of action of **1** and **3** involved COX‐2 inhibition. HMLER‐shEcad cells pre‐treated with lipopolysaccharide (LPS) (2.5 μm for 24 h), to increase basal COX‐2 levels, were treated with **1**, **3**, naproxen, indomethacin, or NiCl_2_⋅6 H_2_O (various concentrations for 72 h) and the COX‐2 expression was determined by flow cytometry. A marked decrease in COX‐2 expression compared to untreated cells was observed for HMLER‐shEcad cells treated with **1** (IC_50_ value for 72 h) and **3** (IC_50_ value for 72 h) (Figure 4 A). As expected, a decrease in COX‐2 expression was also observed in HMLER‐shEcad cells treated with naproxen (20 μm for 72 h) and indomethacin (20 μm for 72 h) (Figure S19). Dosage of HMLER‐shEcad cells with NiCl_2_⋅6 H_2_O (20 μm for 72 h) did not lead to a significant change in the COX‐2 expression (Figure S20). Overall, the flow cytometric data suggests that the cytotoxic mechanism of action of **1** and **3** may involve COX‐2 downregulation. To further prove that **1** and **3** evoke COX‐2‐dependent breast CSC death, cytotoxicity studies were performed with HMLER‐shEcad cells in the presence and absence of prostaglandin E2 (PGE2) (20 μm, 72 h), the product of COX‐2‐mediated arachidonic acid metabolism. The potency of **1** and **3** towards HMLER‐shEcad cells decreased significantly in the presence of PGE2 (IC_50_ value for **1**=29.95(±0.57) μm, 3.9‐fold, *p*<0.05; IC_50_ value for **3**=11.36(±0.33) μm, 6.2‐fold, *p*<0.05) (Figures 4 B, S21), suggesting that **1** and **3** induce breast CSC death through a COX‐2‐dependent pathway.

**Figure 5:**
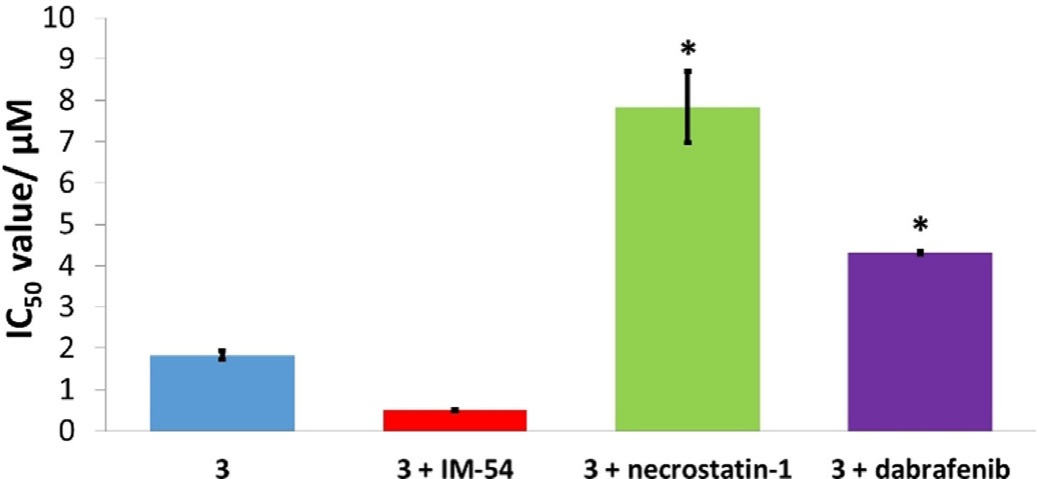
Graphical representation of the IC_50_ values of **3** against HMLER‐shEcad cells in the absence and presence of IM‐54 (10 μm), necrostatin‐1 (20 μm), or dabrafenib (10 μm). Error bars represent standard deviations and Student t‐test, *=*p*<0.05.

Given that we previously reported a nickel(II)‐phenanthroline complex (containing dithiocarbamate ligands) capable of inducing necroptosis in breast CSCs,^[10]^ we investigated the possibility of **1** and **3** to induce random necrosis and programmed necroptosis in breast CSCs. Co‐treatment of **1** and **3** with IM‐54 (10 μm), an inhibitor of unregulated necrosis mediated by oxidative stress,^[24]^ potentiated the toxicity of **1** and **3** towards HMLER‐shEcad cells (IC_50_ value for **1**=6.55(±0.01) μm and IC_50_ value for **3**=0.50(±0.02) μm, Figures 5 and S22–24), indicating that **1** and **3** do not induce unregulated necrosis. Distinct from unregulated necrosis, necroptosis, is a highly ordered form of necrosis that relies on the formation of necrosomes (a protein complex containing RIP1 and RIP3) which initiate cell death.^[25]^ Necroptosis is inhibited by necrostatin‐1 and dabrafenib, small molecule inhibitors of RIP1 and RIP3, respectively.^[26]^ Co‐incubation of **1** and **3** with necrostatin‐1 (20 μm) or dabrafenib (10 μm), significantly (*p*<0.05) reduced the toxicity of **1** and **3** against HMLER‐shEcad cells (IC_50_ value for **1**=13.09(±0.33) μm with necrostatin‐1, and 10.63(±0.92) μm with dabrafenib; IC_50_ value for **3**=7.84±0.86 μm with necrostatin‐1, and 4.33(±0.04) μm with dabrafenib, Figures 5 and S22–24). A similar attenuation in potency was observed for shikonin (an established necroptosis inducer) in the presence of necrostatin‐1 or dabrafenib under identical conditions.^[10]^ Collectively this suggests that **1** and **3** can induce necroptotic breast CSC death.

## Conclusions

In summary, we report two nickel(II)‐3,4,7,8‐tetramethyl‐1,10‐phenanthroline complexes bearing NSAID moieties (naproxen and indomethacin), **1** and **3** capable of killing bulk breast cancer cells and breast CSC in the micromolar range (under in vitro monolayer conditions). Notably, **1** and **3** exhibited significantly lower toxicity towards non‐cancerous epithelial breast cells than breast CSCs or bulk breast cancer cells (up to 4.6‐fold). The indomethacin‐containing nickel(II) complex, **3** displayed significantly greater potency for breast CSCs than salinomycin and cisplatin in monolayer systems. Furthermore, **3** inhibited the formation and size of breast CSC mammospheres to a similar extent as salinomycin, in three‐dimensional cell culture conditions. Previous work has shown that the analogous copper(II) complexes, **Cu‐1** (with naproxen) and **Cu‐3** (with indomethacin) displayed sub‐micromolar IC_50_ values towards breast CSCs and bulk breast cancer cells, which is significantly lower than the IC_50_ values observed for **1** and **3**.^[8b]^ Interestingly, **Cu‐1** killed breast CSCs with higher potency than bulk breast cancer cells (akin to **1** and **3**), whereas **Cu‐3** displayed the opposite trend. Taken together, this suggests that within the metal(II)‐phenanthroline‐NSAIDs family of compounds, breast CSC and bulk breast cancer cell potency and selectivity (with respect to each other) can be modulated by altering the metal, phenanthroline‐based ligand, or NSAID components.

Mechanistic studies indicated that **1** and **3** reduced the overall expression of COX‐2 in breast CSCs and induced breast CSC death through a COX‐2‐dependent pathway. Additionally, the cytotoxicity of **1** and **3** toward breast CSCs was significantly reduced in the presence of necroptosis inhibitors (necrostatin‐1 and dabrafenib), suggesting that **1** and **3** can induce necroptotic breast CSC death. The widespread use of conventional apoptosis‐inducing anticancer agents has led to high incidences of apoptosis resistance in tumours. One of the reasons for apoptosis resistance is the presence of CSCs within tumours. CSCs inherently possess ineffective or incomplete apoptosis signalling pathways. Therefore, compounds such as **1** and **3**, which can potentially evoke cell death via non‐apoptotic pathways, could hold the key to overcoming apoptosis resistant CSCs. Our findings could pave the way for the development of other necroptosis‐inducing, COX‐inhibiting compounds for CSC‐directed chemotherapy.

## Experimental Section

### Materials and methods

All synthetic procedures were performed under normal atmospheric conditions or under nitrogen. Fourier transform infrared (FTIR) spectra were recorded with an IRAffinity‐1S Shimadzu spectrophotometer. UV/Vis absorption spectra were recorded on a Cary 3500 UV/Vis spectrophotometer. Elemental analysis of the compounds prepared was performed commercially by London Metropolitan University. NiCl_2_⋅6 H_2_O, 3,4,7,8‐tetramethyl‐1,10‐phenanthroline, and 4,7‐diphenyl‐1,10‐phenanthroline were purchased from Sigma Aldrich and used as received.

### Synthesis and characterisation

**Synthesis of [Ni(naproxen‐*O*)(naproxen‐*O,O’*)(3,4,7,8‐tetramethyl‐1,10‐phenanthroline)(H_2_O)] (1)**: A methanolic solution (10 mL) of naproxen (93 mg, 0.4 mmol) and KOH (24 mg, 0.4 mmol) was stirred for 30 min. To the resulting solution, a methanolic solution (5 mL) of NiCl_2_⋅6 H_2_O (48 mg, 0.2 mmol) was added simultaneously with a methanolic solution (5 mL) of 3,4,7,8‐tetramethyl‐1,10‐phenanthroline (49 mg, 0.2 mmol). The resulting solution was stirred for another 30 min before being fully dried. The resulting solid was washed with water (10 mL) and diethyl ether (10 mL) to yield **1** as a pale green powder (117 mg, 77 %). UV (chloroform, nm): 277, 304, 332; ATR‐FTIR (solid, cm^−1^): 2971, 1603, 1546, 1526, 1437, 1390, 1264, 1213, 1162, 1030, 928, 887, 858, 806, 724,693, 622, 531; Anal. calcd. for **1**, C_44_H_44_N_2_NiO_7_⋅0.5H_2_O (%): C, 67.71; H, 5.81; N, 3.59. Found: C, 67.41; H, 5.71; N, 3.26.

**Synthesis of [Ni(naproxen‐*O*)(naproxen‐*O,O’*)(4,7‐diphenyl‐1,10‐phenanthroline)(H_2_O)] (2)**: A methanolic solution (10 mL) of naproxen (92 mg, 0.4 mmol) and KOH (26 mg, 0.5 mmol) was stirred for 30 min. To the resulting solution, a methanolic solution (5 mL) of NiCl_2_⋅6 H_2_O (49 mg, 0.2 mmol) was added simultaneously with a methanolic solution (10 mL) of 4,7‐diphenyl‐1,10‐phenanthroline (68 mg, 0.2 mmol). The resulting solution was stirred for another 30 min before being fully dried. The resulting solid was washed with water (10 mL) and diethyl ether (10 mL) to yield **2** as a pale green powder (121 mg, 69 %). UV (chloroform, nm): 282, 319, 357; ATR‐FTIR (solid, cm^−1^): 2976, 1605, 1561, 1520, 1391, 1265, 1214, 1164, 1031, 929, 858, 817, 766, 746, 705, 634, 573, 552; Anal. calcd. for **2**, C_52_H_44_N_2_NiO_7_ (%): C, 71.99; H, 5.11; N, 3.23. Found: C, 72.26; H, 5.38; N, 3.45.

**Synthesis of [Ni(indomethacin‐*O*)_2_(3,4,7,8‐tetramethyl‐1,10‐phenanthroline)(H_2_O)_2_] (3)**: A methanolic solution (10 mL) of indomethacin (143 mg, 0.4 mmol) and KOH (22 mg, 0.4 mmol) was stirred for 1 h. To the resulting solution, a methanolic solution (5 mL) of NiCl_2_⋅6 H_2_O (48 mg, 0.2 mmol) was added simultaneously with a methanolic solution (5 mL) of 3,4,7,8‐tetramethyl‐1,10‐phenanthroline (47 mg, 0.2 mmol). The resulting solution was stirred for another 30 min before being fully dried. The resulting solid was washed with water (10 mL) and diethyl ether (10 mL) to yield **3** as a green powder (122 mg, 60 %). UV (chloroform, nm): 275, 304, 329; ATR‐FTIR (solid, cm^−1^): 2935, 1681, 1590, 1478, 1437, 1355, 1315, 1223, 1142, 1081, 1019, 918, 836, 755, 724, 602, 551; Anal. calcd. for **3**, C_54_H_50_Cl_2_N_4_NiO_10_⋅0.5H_2_O (%): C, 61.56; H, 4.88; N, 5.32. Found: C, 61.85; H, 4.74; N, 5.07.

**Synthesis of [Ni(indomethacin‐*O*)_2_(4,7‐diphenyl‐1,10‐phenanthroline)(H_2_O)_2_] (4)**: A methanolic solution (10 mL) of indomethacin (147 mg, 0.4 mmol) and KOH (23 mg, 0.4 mmol) was stirred for 1 h. To the resulting solution, a methanolic solution (5 mL) of NiCl_2_⋅6 H_2_O (49 mg, 0.2 mmol) was added simultaneously with a methanolic solution (5 mL) of 4,7‐diphenyl‐1,10‐phenanthroline (67 mg, 0.2 mmol). The resulting solution was stirred for another 30 min before being fully dried. The resulting solid was washed with water (10 mL) and diethyl ether (10 mL) to yield **4** as a green powder (205 mg, 90 %). UV (chloroform, nm): 281, 320, 358; ATR‐FTIR (solid, cm^−1^): 2928, 1677, 1594, 1555, 1474, 1402, 1356, 1321, 1230, 1148, 1087, 1026, 924, 824, 761, 700, 669, 639, 598, 547; Anal. calcd. for **4**, C_62_H_50_Cl_2_N_4_NiO_10_ (%): C, 65.28; H, 4.42; N, 4.91. Found: C, 65.65; H, 4.61; N, 4.90.

### Measurement of water‐octanol partition coefficient (LogP)

The LogP value for **1**–**4** was determined using the shake‐flask method and UV/Vis spectroscopy. The octanol used in this experiment was pre‐saturated with water. An aqueous solution (pH 6.99) of **1**–**4** (500 μL, 100 μm) was incubated with octanol (500 μL) in a 1.5 mL tube. The tube was shaken at room temperature for 24 h. The two phases were separated by centrifugation and the **1**–**4** content in each phase was determined by UV/Vis spectroscopy.

### Cell lines and cell culture conditions

The human mammary epithelial cell lines, HMLER and HMLER‐shEcad were kindly donated by Prof. R. A. Weinberg (Whitehead Institute, MIT). The human epithelial breast MCF710A cell line was acquired from the American Type Culture Collection (ATCC, Manassas, VA, USA). HMLER, HMLER‐shEcad, and MCF10A cells were maintained in Mammary Epithelial Cell Growth Medium (MEGM) with supplements and growth factors (BPE, hydrocortisone, hEGF, insulin, and gentamicin/amphotericin‐B). The cells were grown at 310 K in a humidified atmosphere containing 5 % CO_2_.

### Cytotoxicity MTT assay

The colorimetric MTT assay was used to determine the toxicity of **1**, **3**, and NiCl_2_⋅6 H_2_O. HMLER, HMLER‐shEcad, and MCF10A cells (5×10^3^) were seeded in each well of a 96‐well plate. After incubating the cells overnight, various concentrations of the compounds (0.2–100 μm), were added and incubated for 72 h (total volume 200 μL). Stock solutions of the compounds were prepared as 10 mm solutions in DMSO and diluted using media. The final concentration of DMSO in each well was 0.5 % and this amount was present in the untreated control as well. After 72 h, 20 μL of a 4 mg mL^−1^ solution of MTT in PBS was added to each well, and the plate was incubated for an additional 4 h. The MEGM/MTT mixture was aspirated and 200 μL of DMSO was added to dissolve the resulting purple formazan crystals. The absorbance of the solutions in each well was read at 550 nm. Absorbance values were normalized to (DMSO‐containing) control wells and plotted as concentration of test compound versus % cell viability. IC_50_ values were interpolated from the resulting dose dependent curves. The reported IC_50_ values are the average of three independent experiments (*n*=12).

### Tumoursphere formation and viability assay

HMLER‐shEcad cells (5×10^3^) were plated in ultralow‐attachment 96‐well plates (Corning) and incubated in MEGM supplemented with B27 (Invitrogen), 20 ng mL^−1^ EGF, and 4 μg mL^−1^ heparin (Sigma) for 5 days. Studies were also conducted in the presence of **1**, **3**, salinomycin, NiCl_2_⋅6 H_2_O, and naproxen (0–133 μm). Mammospheres treated with **1**, **3**, NiCl_2_⋅6 H_2_O, and salinomycin (at their respective IC_20_ values, 5 days) were counted and imaged using an inverted microscope. The viability of the mammospheres was determined by addition of a resazurin‐based reagent, TOX8 (Sigma). After incubation for 16 h, the fluorescence of the solutions was read at 590 nm (*λ*_ex_=560 nm). Viable mammospheres reduce the amount of the oxidized TOX8 form (blue) and concurrently increases the amount of the fluorescent TOX8 intermediate (red), indicating the degree of mammosphere cytotoxicity caused by the test compound. Fluorescence values were normalized to DMSO‐containing controls and plotted as concentration of test compound versus % mammospheres viability. IC_50_ values were interpolated from the resulting dose dependent curves. The reported IC_50_ values are the average of three independent experiments, each consisting of two replicates per concentration level (overall *n*=6).

### Cellular uptake

To measure the cellular uptake of **1** and **3**, about 1 million HMLER‐shEcad cells were treated with **1** or **3** (at 5 μm) at 37 °C for 24 h. After incubation, the media was removed, the cells were washed with PBS (2 mL × 3), and harvested. The number of cells was counted at this stage, using a haemocytometer. This mitigates any cell death induced by **1** and **3** at the administered concentration and experimental cell loss. The cellular pellets were dissolved in 65 % HNO_3_ (250 μL) overnight. Cellular pellets of **1** and **3** treated HMLER‐shEcad cells were also used to determine the nickel content in the nuclear, cytoplasmic, and membrane fractions. The Thermo Scientific NE‐PER Nuclear and Cytoplasmic Extraction Kit was used to extract and separate the nuclear, cytoplasmic, and membrane fractions. The fractions were dissolved in 65 % HNO_3_ overnight (250 μL final volume). All samples were diluted fivefold with water and analysed using inductively coupled plasma mass spectrometry (ICP‐MS, ThermoScientific ICAP‐Qc quadrupole ICP mass spectrometer). Nickel levels are expressed as Ni (ppb) per million cells. Results are presented as the mean of four determinations for each data point.

### Flow cytometry

HMLER‐shEcad cells were seeded in 6‐well plates (at a density of 5×10^5^ cells per mL) and the cells were allowed to attach overnight. The cells were treated with lipopolysaccharide (LPS) (2.5 μg L^−1^ for 24 h), and then treated with **1** (IC_50_ value), **3** (IC_50_ value), naproxen (20 μm), indomethacin (20 μm), or NiCl_2_⋅6 H_2_O (20 μm) and incubated for a further 72 h. The cells were then harvested by trypsinization, fixed with 4 % paraformaldehyde (at 37 °C for 10 min), permeabilized with ice‐cold methanol (for 30 min), and suspended in PBS (200 μL). The Alexa Fluor® 488 nm labelled anti‐COX‐2 antibody (5 μL) was then added to the cell suspension and incubated in the dark for 1 h. The cells were then washed with PBS (1 mL) and analysed using a FACSCanto II flow cytometer (BD Biosciences) (10,000 events per sample were acquired) at the University of Leicester FACS Facility. The FL1 channel was used to assess COX‐2 expression. Cell populations were analysed using the FlowJo software (Tree Star).

## Conflict of interest

The authors declare no conflict of interest.

## Supporting information

As a service to our authors and readers, this journal provides supporting information supplied by the authors. Such materials are peer reviewed and may be re‐organized for online delivery, but are not copy‐edited or typeset. Technical support issues arising from supporting information (other than missing files) should be addressed to the authors.

Supplementary
