# Supplementary material for: Breast Cancer Stem Cell Potency of Nickel(II)‐Polypyridyl Complexes Containing Non‐steroidal Anti‐inflammatory Drugs
Source: Chemistry. 2020 Sep 30;26(61):14011–7. doi: 10.1002/chem.202001578 (PMC7702150; doi:10.1002/chem.202001578)
Supplement: Supplementary file 1 — Supplementary [file CHEM-26-14011-s001.pdf]

Chemistry–A European Journal

Supporting Information

Breast Cancer Stem Cell Potency of Nickel(II)-Polypyridyl Complexes Containing Non-steroidal Anti-inflammatory Drugs

Catherine J. Feld+, Alice Johnson+, Zhiyin Xiao, and Kogularamanan Suntharalingam*[a]

<u>Table of Content</u>

**Figure S1.**	ATR-FTIR spectra of (A) **1**, (B) **2**, (C) **3**, and (D) **4** in the solid form.
**Figure S2.**	UV-Vis spectra of **1-4** (50 μM) in chloroform at room temperature.
**Table S1.**	Experimentally determined LogP values for **1-4**.
**Figure S3.**	UV-Vis spectrum of **1** (50 μM) in PBS:DMSO (200:1) over the course of 24 h at 37 °C.
**Figure S4.**	UV-Vis spectrum of **3** (50 μM) in PBS:DMSO (200:1) over the course of 24 h at 37 °C.
**Figure S5.**	UV-Vis spectrum of **2** (50 μM) in PBS:DMSO (200:1) over the course of 24 h at 37 °C.
**Figure S6.**	UV-Vis spectrum of **4** (50 μM) in PBS:DMSO (200:1) over the course of 24 h at 37 °C.
**Figure S7.**	UV-Vis spectrum of **1** in mammary epithelial cell growth medium (MEGM):DMSO (200:1) over the course of 24 h at 37 °C.
**Figure S8.**	UV-Vis spectrum of **3** in mammary epithelial cell growth medium (MEGM):DMSO (200:1) over the course of 24 h at 37 °C.
**Figure S9.**	UV-Vis spectrum of **2** in mammary epithelial cell growth medium (MEGM):DMSO (200:1) over the course of 24 h at 37 °C.
**Figure S10.**	UV-Vis spectrum of **4** in mammary epithelial cell growth medium (MEGM):DMSO (200:1) over the course of 24 h at 37 °C.
**Figure S11.**	Representative dose-response curves for the treatment of HMLER and HMLER-shEcad cells with **1**.
**Figure S12.**	Representative dose-response curves for the treatment of HMLER and HMLER-shEcad cells with **3**.
**Figure S13.**	Chemical structures of the copper(II)-3,4,7,8-tetramethyl-1,10-phenanthroline complexes containing the non-steroidal anti-inflammatory drugs (NSAIDs), naproxen and indomethacin, **Cu-1** and **Cu-3**.
**Figure S14.**	Representative dose-response curves for the treatment of HMLER and HMLER-shEcad cells with $NiCl_2 \cdot 6H_2O$.
**Figure S15.**	Representative dose-response curves for the treatment of MCF10A cells with **1** and **3**.
**Figure S16.**	Quantification of mammosphere formation with HMLER-shEcad cells untreated and treated with $NiCl_2 \cdot 6H_2O$ (at 2 μM for 5 days). Error bars = SD.
**Figure S17.**	Representative bright-field images (x 10) of the mammospheres in the absence and presence of $NiCl_2 \cdot 6H_2O$ (at 2 μM for 5 days).
**Figure S18.**	Representative dose-response curves for the treatment of HMLER-shEcad mammospheres with **1**, **3**, $NiCl_2 \cdot 6H_2O$, or naproxen after 5 days incubation.
**Figure S19.**	Representative histograms displaying the green fluorescence emitted by anti-COX-2 Alexa Fluor 488 nm antibody-stained HMLER-shEcad cells treated with LPS (2.5 μM) for 24 h (red) followed by 72 h in media containing naproxen (20 μM, blue) or indomethacin (20 μM, orange).
**Figure S20.**	Representative histograms displaying the green fluorescence emitted by anti-COX-2 Alexa Fluor 488 nm antibody-stained HMLER-shEcad cells treated with LPS (2.5 μM) for 24 h (red) followed by 72 h in media containing $NiCl_2 \cdot 6H_2O$ (20 μM, blue).

Figure S21. Representative dose-response curves for the treatment of HMLER-shEcad cells with **1** after 72 incubation in the presence and absence of PGE2 (20 μM).

Figure S22. Representative dose-response curves for the treatment of HMLER-shEcad cells with **1** after 72 incubation in the presence and absence of IM-54 (10 μM), necrostatin-1 (20 μM), or dabrafenib (20 μM).

Figure S23. Representative dose-response curves for the treatment of HMLER-shEcad cells with **3** after 72 incubation in the presence and absence of IM-54 (10 μM), necrostatin-1 (20 μM), or dabrafenib (20 μM).

Figure S24. Graphical representation of the IC_{50} values of **1** against HMLER-shEcad cells in the absence and presence of IM-54 (10 μM), necrostatin-1 (20 μM), or dabrafenib (10 μM). Error bars represent standard deviations and Student t-test, * = $p < 0.05$.

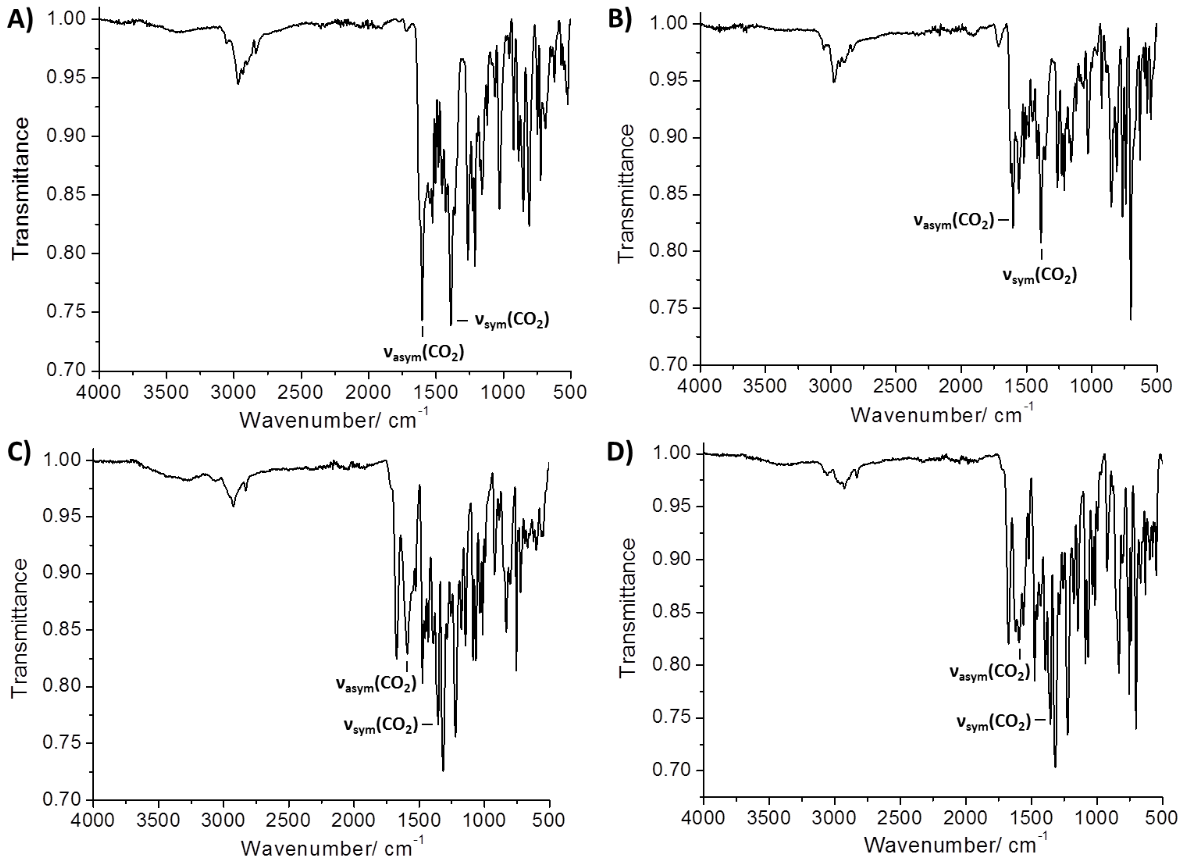

Figure S1. ATR-FTIR spectra of (A) **1**, (B) **2**, (C) **3**, and (D) **4** in the solid form.

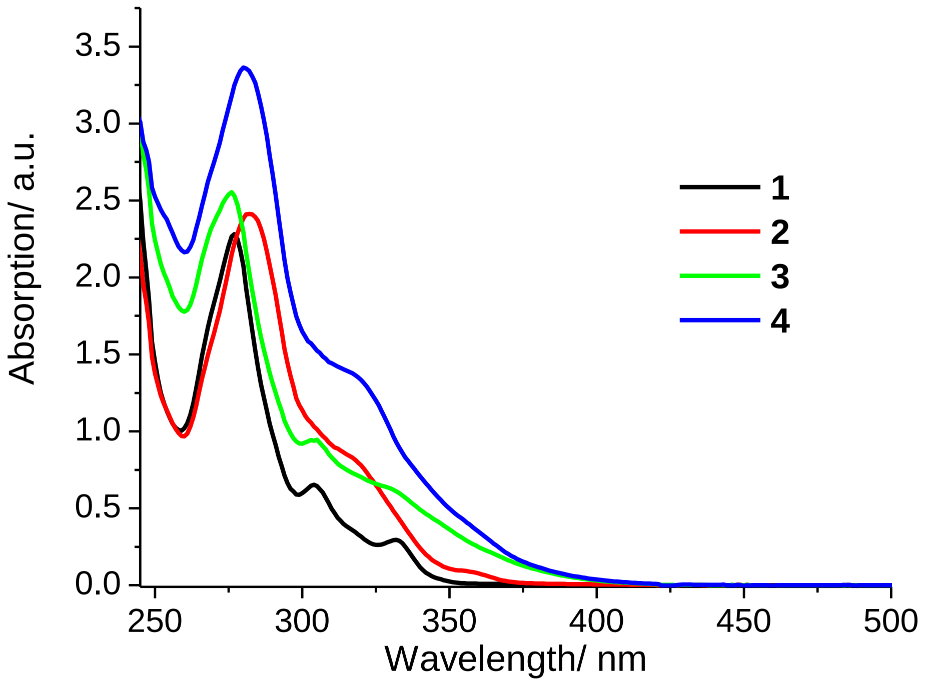

Figure S2. UV-Vis spectra of **1-4** (50 μM) in chloroform at room temperature.

Table S1. Experimentally determined LogP values for **1-4**.

Metal complex	LogP
**1**	0.63 ± 0.05
**2**	1.21 ± 0.11
**3**	0.96 ± 0.13
**4**	1.52 ± 0.14

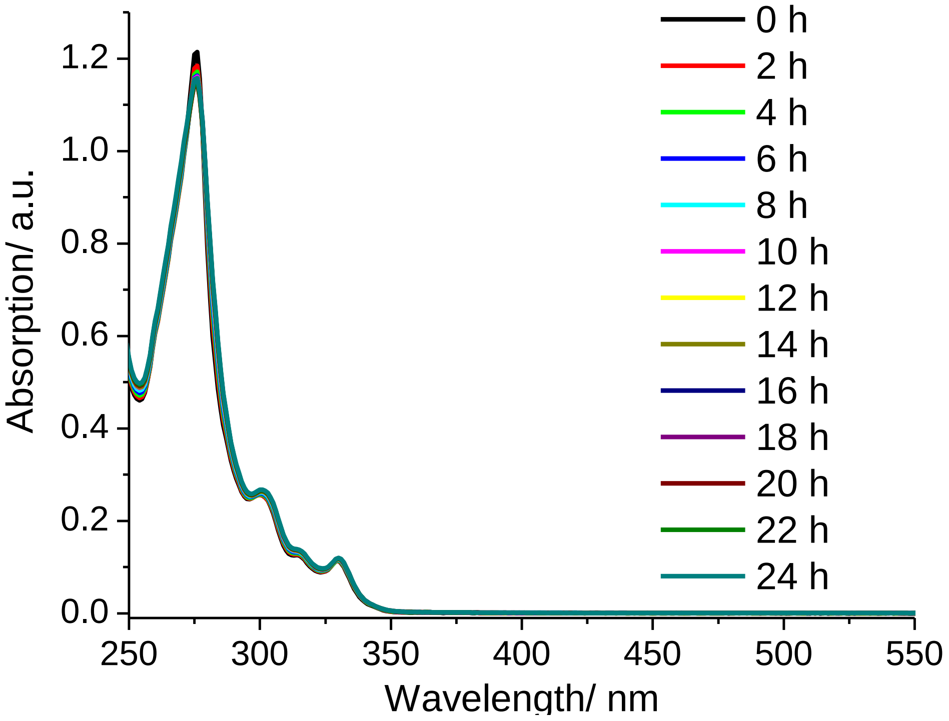

Figure S3. UV-Vis spectrum of **1** (50 µM) in PBS:DMSO (200:1) over the course of 24 h at 37 °C.

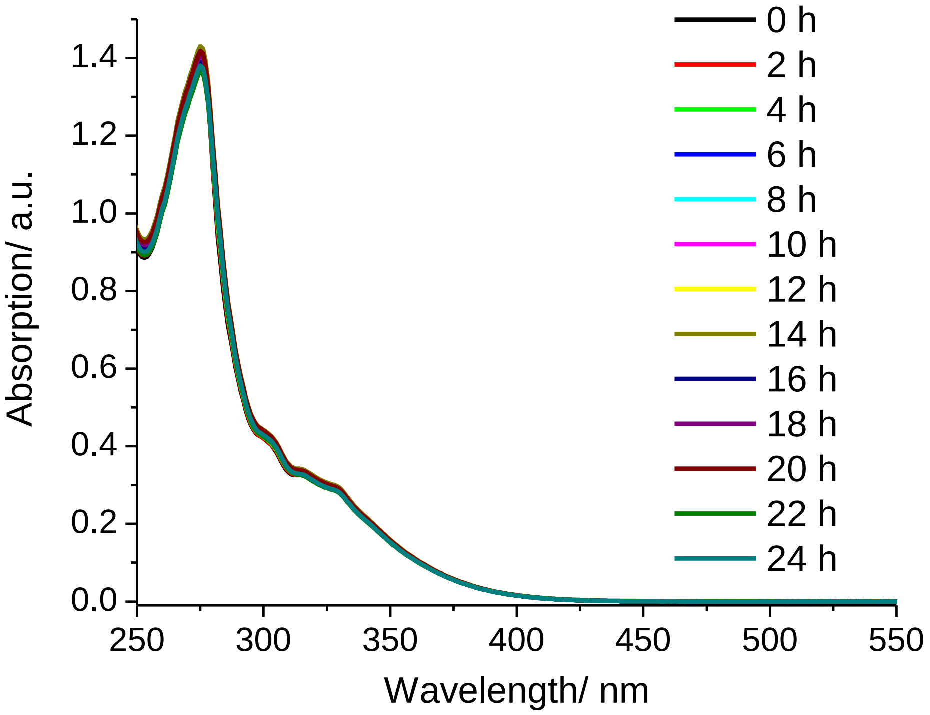

Figure S4. UV-Vis spectrum of **3** (50 µM) in PBS:DMSO (200:1) over the course of 24 h at 37 °C.

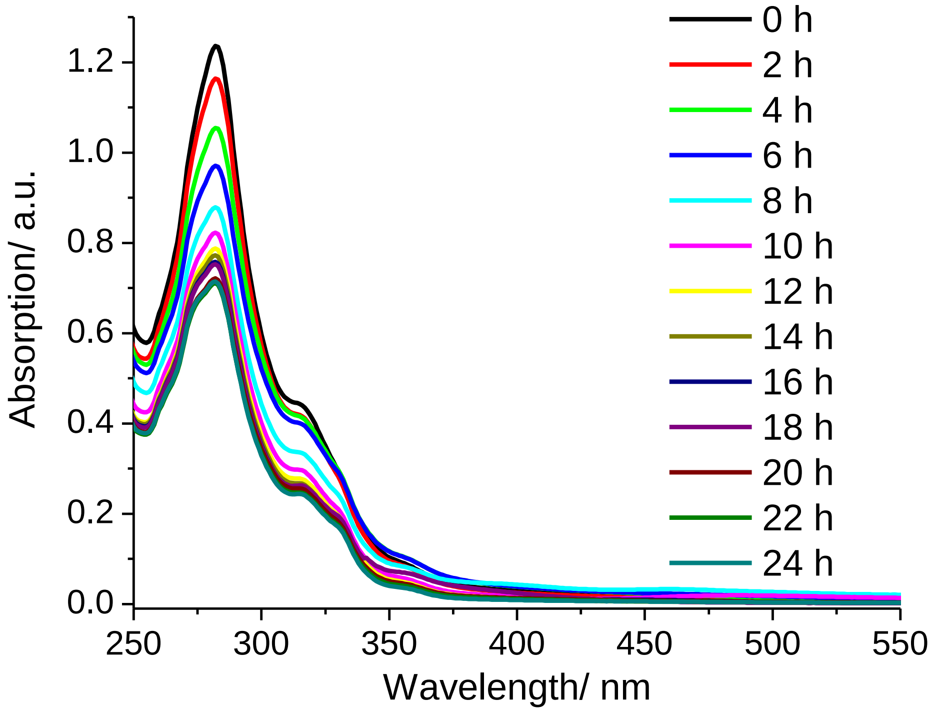

Figure S5. UV-Vis spectrum of **2** (50 µM) in PBS:DMSO (200:1) over the course of 24 h at 37 °C.

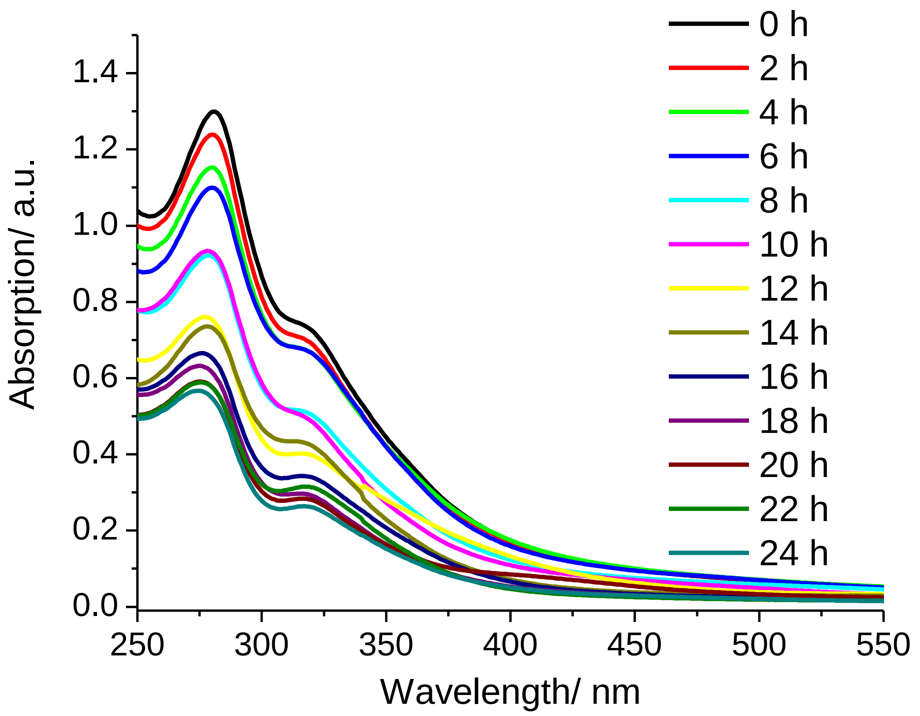

Figure S6. UV-Vis spectrum of **4** (50 µM) in PBS:DMSO (200:1) over the course of 24 h at 37 °C.

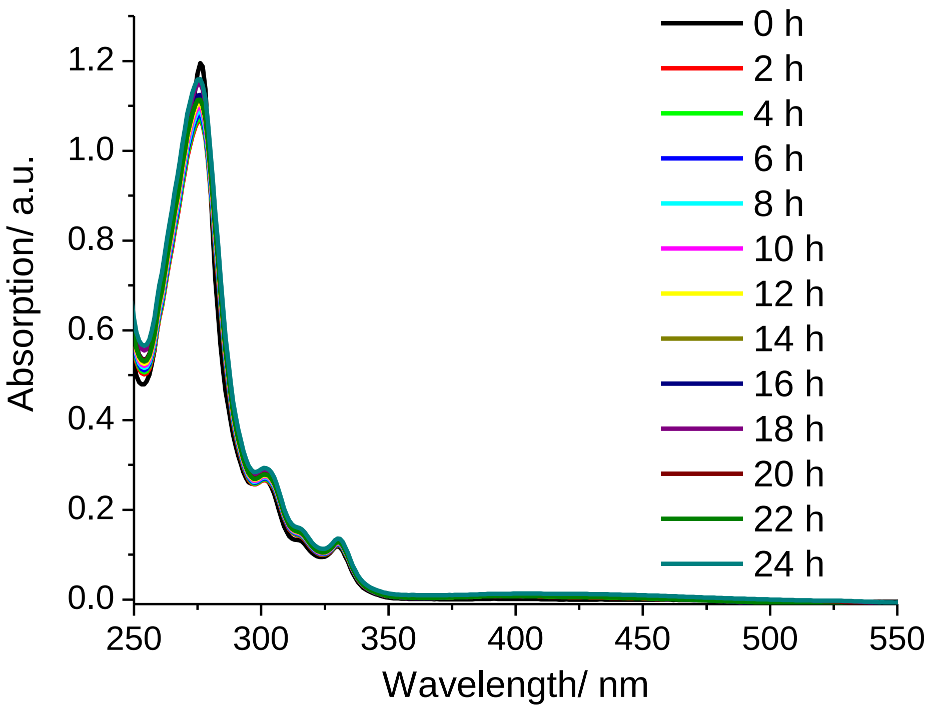

Figure S7. UV-Vis spectrum of **1** in mammary epithelial cell growth medium (MEGM):DMSO (200:1) over the course of 24 h at 37 °C.

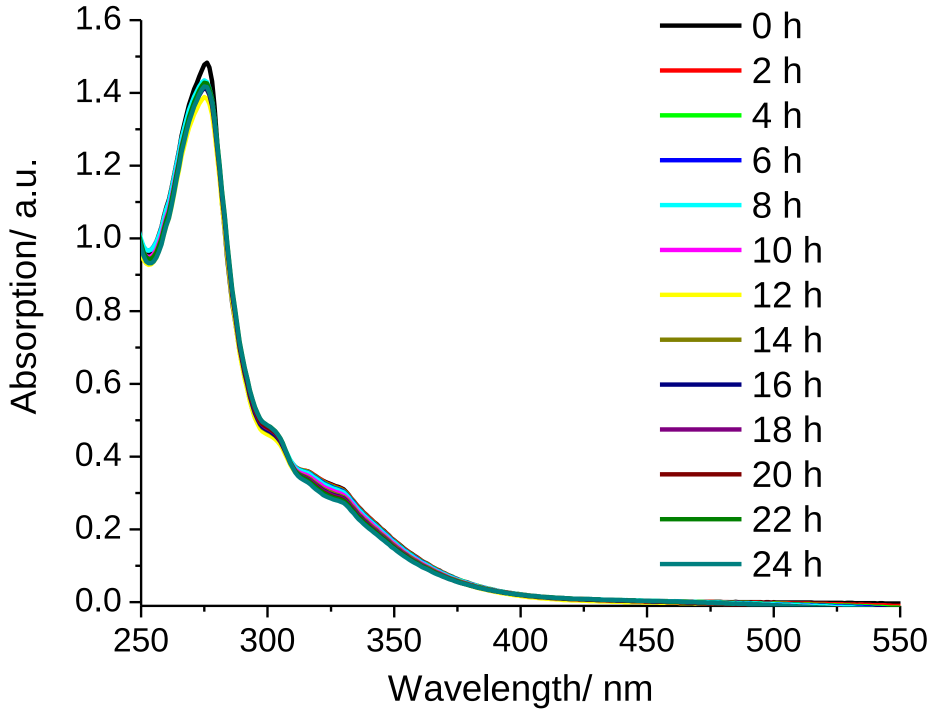

Figure S8. UV-Vis spectrum of **3** in mammary epithelial cell growth medium (MEGM):DMSO (200:1) over the course of 24 h at 37 °C.

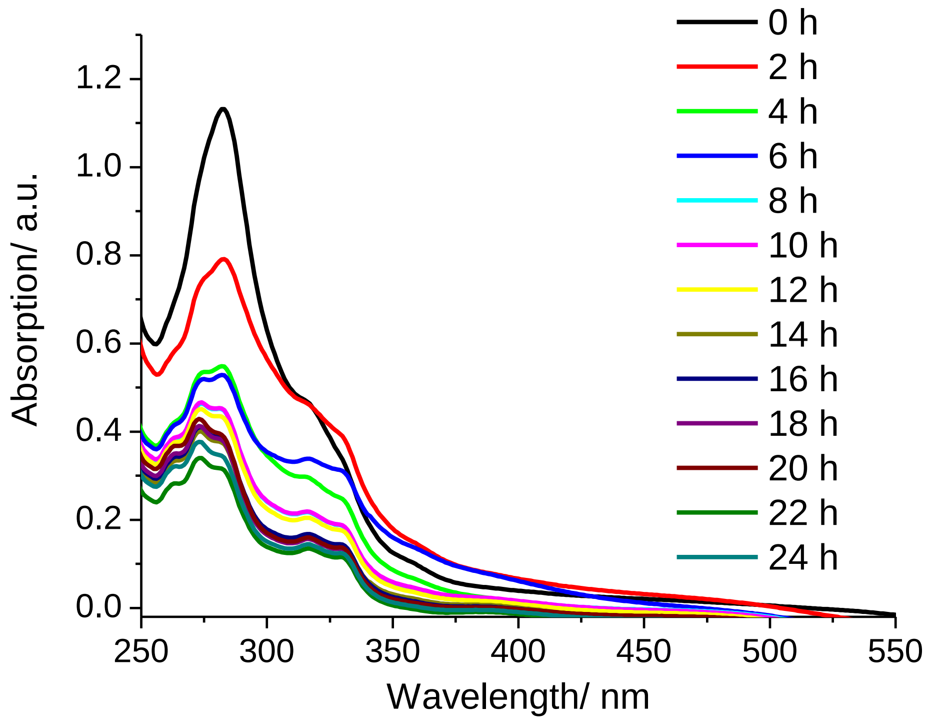

Figure S9. UV-Vis spectrum of **2** in mammary epithelial cell growth medium (MEGM):DMSO (200:1) over the course of 24 h at 37 °C.

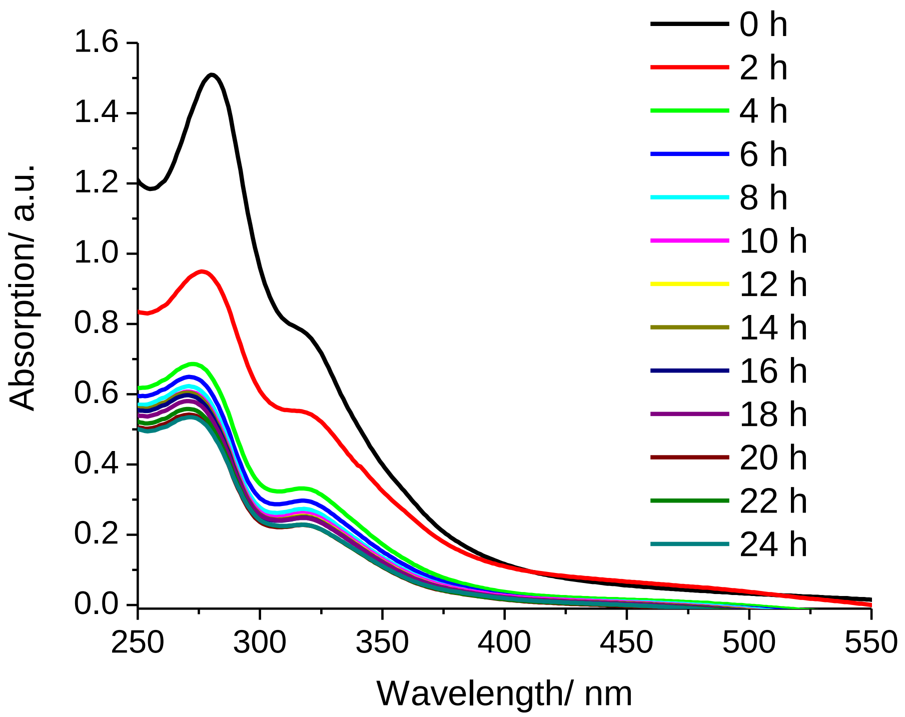

Figure S10. UV-Vis spectrum of **4** in mammary epithelial cell growth medium (MEGM):DMSO (200:1) over the course of 24 h at 37 °C.

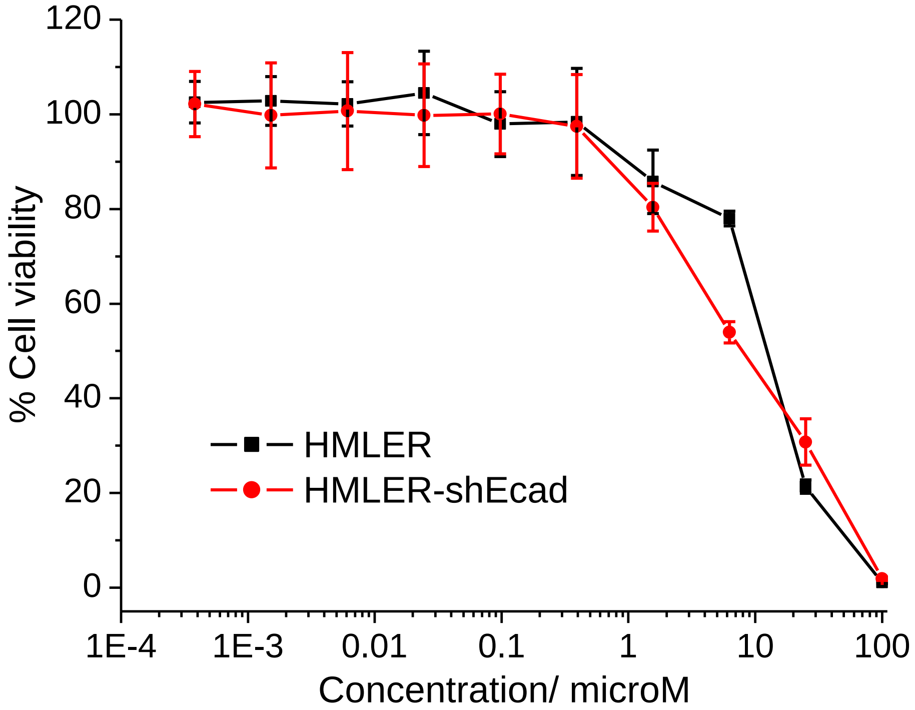

Figure S11. Representative dose-response curves for the treatment of HMLER and HMLER-shEcad cells with **1**.

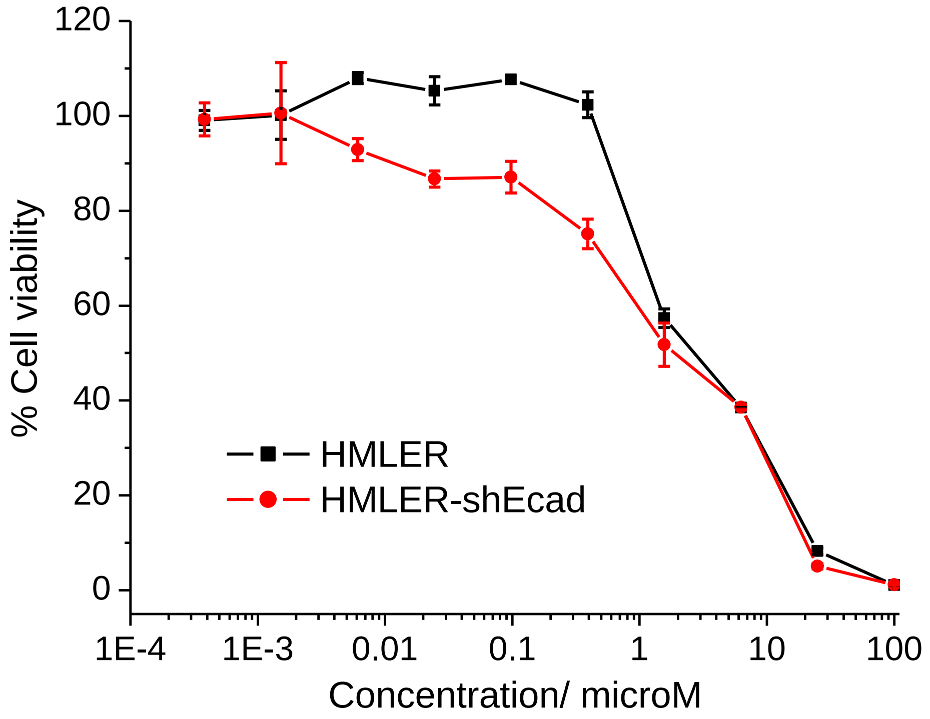

Figure S12. Representative dose-response curves for the treatment of HMLER and HMLER-shEcad cells with **3**.

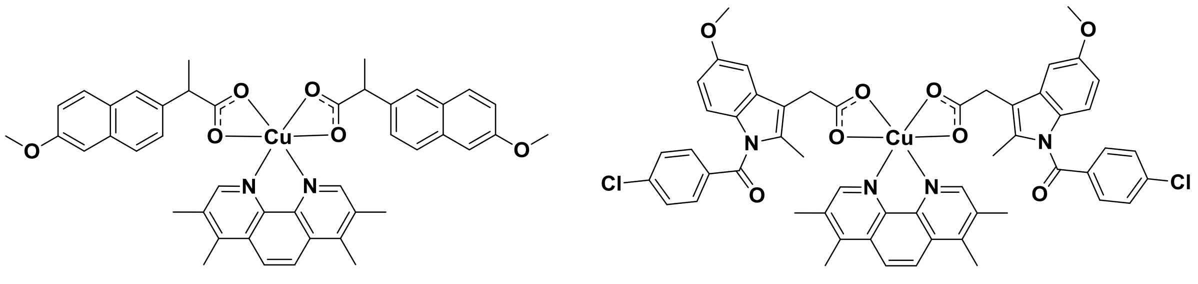

Figure S13. Chemical structures of the copper(II)-3,4,7,8-tetramethyl-1,10-phenanthroline complexes containing the non-steroidal anti-inflammatory drugs (NSAIDs), naproxen and indomethacin, **Cu-1** and **Cu-3**.

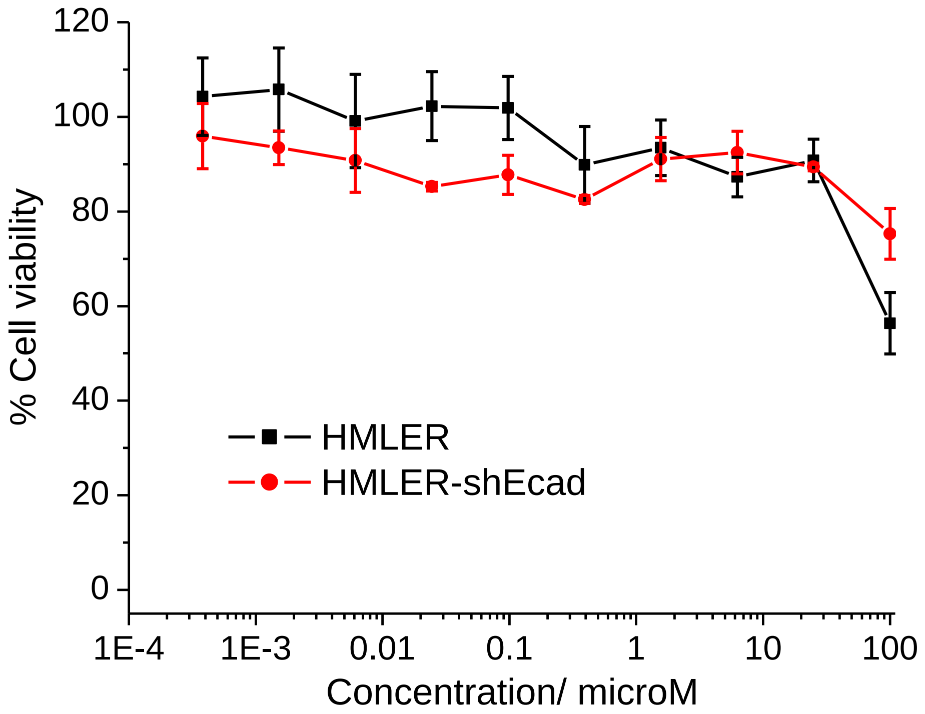

Figure S14. Representative dose-response curves for the treatment of HMLER and HMLER-shEcad cells with $NiCl_2 \cdot 6H_2O$.

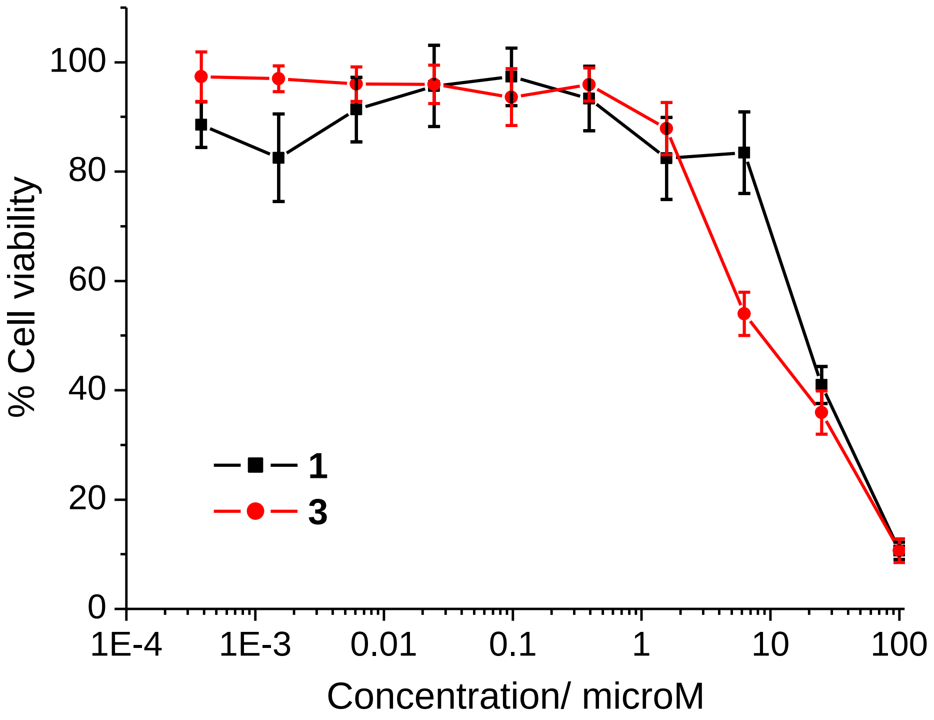

Figure S15. Representative dose-response curves for the treatment of MCF10A cells with **1** and **3**.

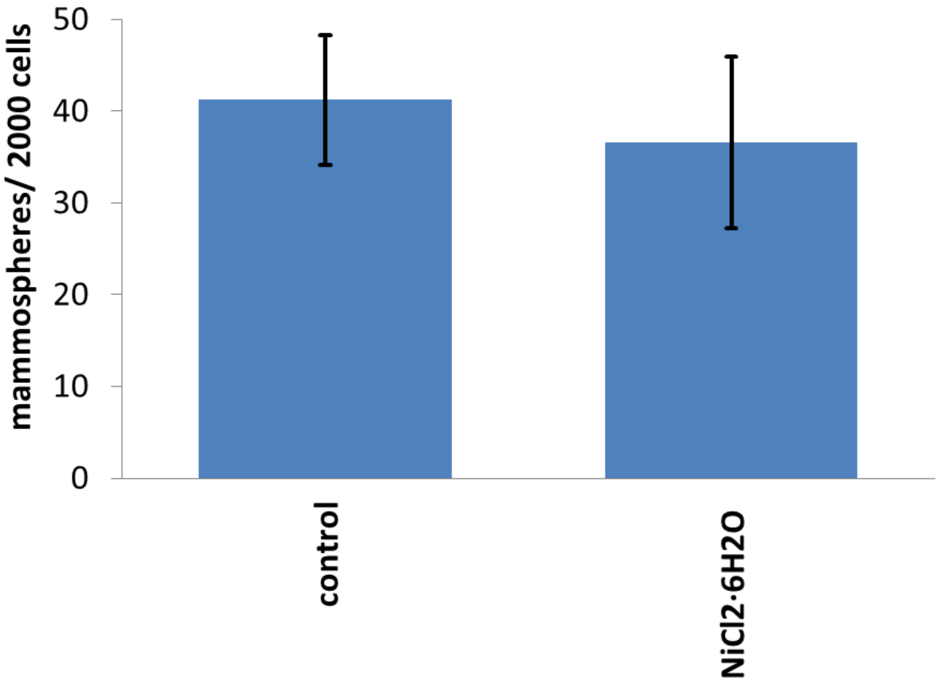

Figure S16. Quantification of mammosphere formation with HMLER-shEcad cells untreated and treated with $NiCl_2{\cdot}6H_2O$ (at 2 µM for 5 days). Error bars = SD.

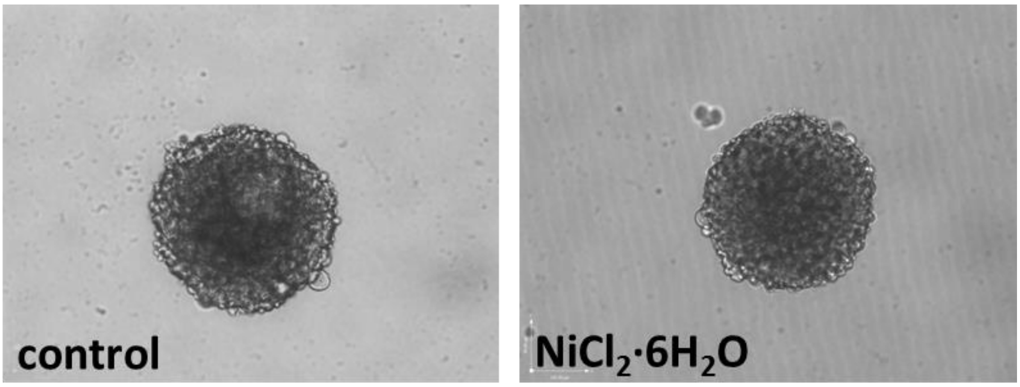

Figure S17. Representative bright-field images (x 10) of the mammospheres in the absence and presence of $NiCl_2{\cdot}6H_2O$ (at 2 µM for 5 days).

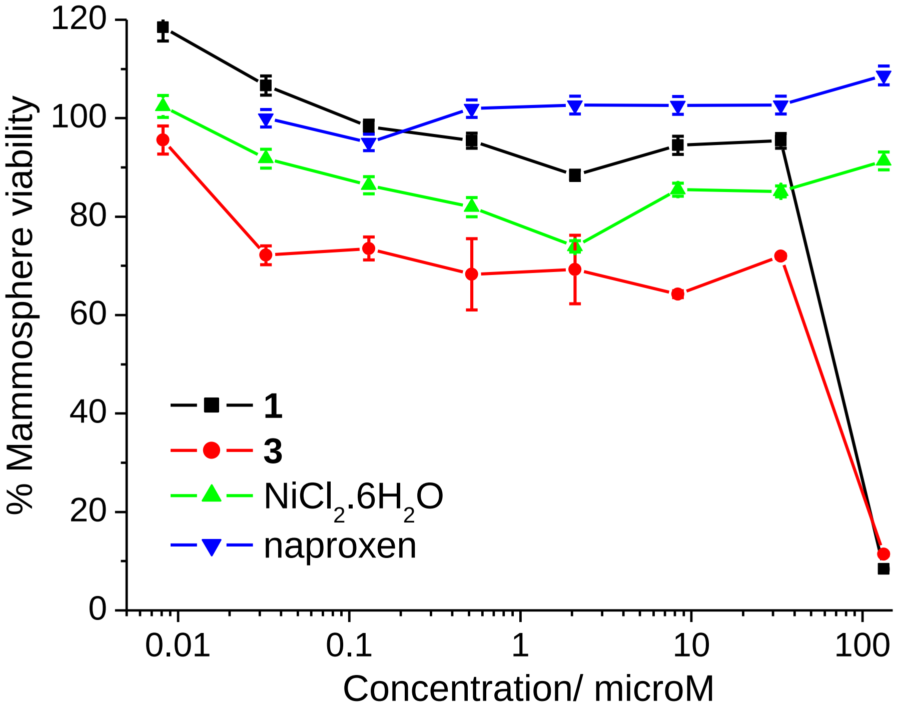

Figure S18. Representative dose-response curves for the treatment of HMLER-shEcad mammospheres with **1**, **3**, $NiCl_2{\cdot}6H_2O$, or naproxen after 5 days incubation.

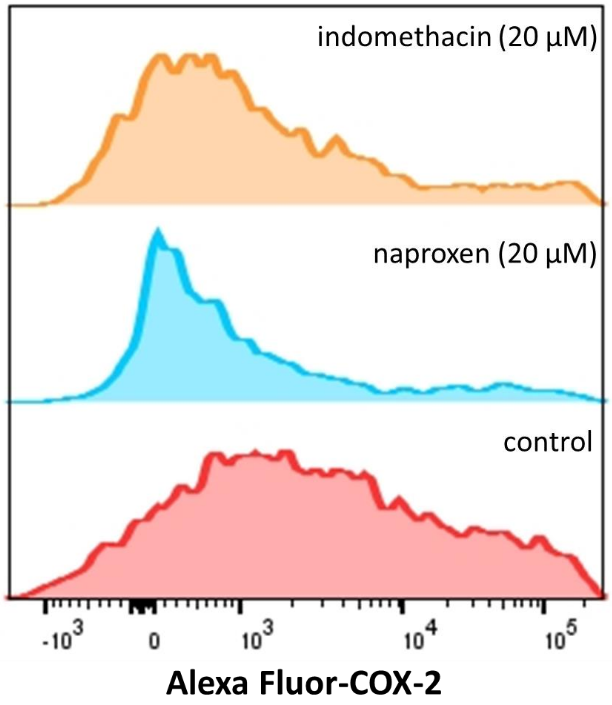

Figure S19. Representative histograms displaying the green fluorescence emitted by anti-COX-2 Alexa Fluor 488 nm antibody-stained HMLER-shEcad cells treated with LPS (2.5 µM) for 24 h (red) followed by 72 h in media containing naproxen (20 µM, blue) or indomethacin (20 µM, orange).

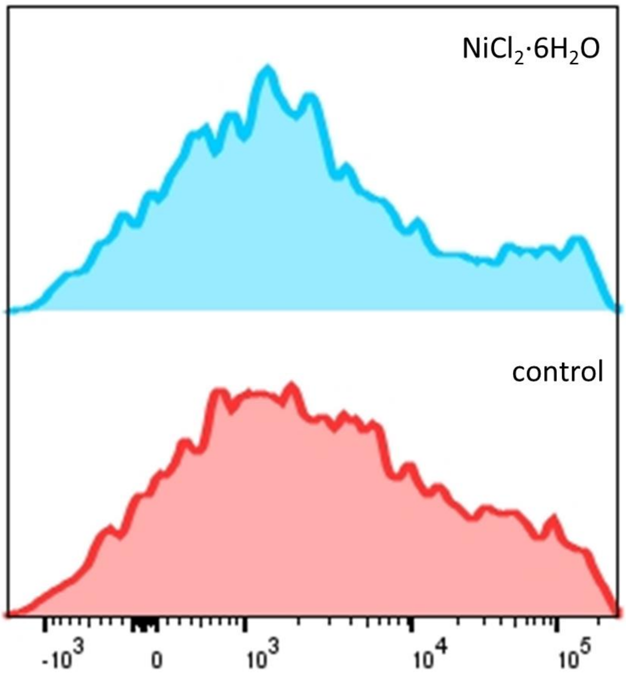

Figure S20. Representative histograms displaying the green fluorescence emitted by anti-COX-2 Alexa Fluor 488 nm antibody-stained HMLER-shEcad cells treated with LPS (2.5 µM) for 24 h (red) followed by 72 h in media containing $NiCl_2{\cdot}6H_2O$ (20 µM, blue).

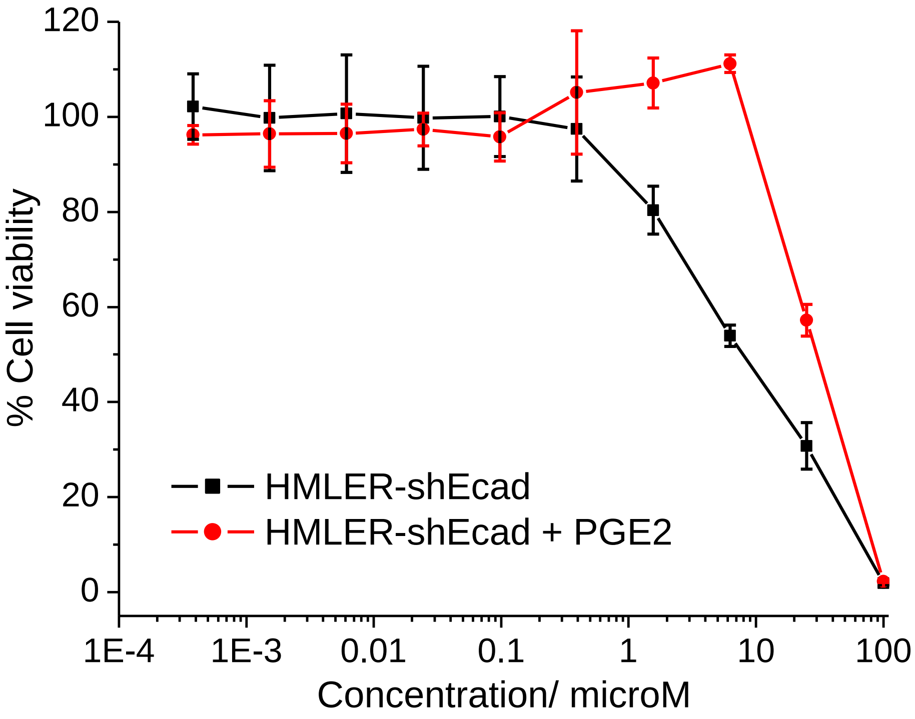

Figure S21. Representative dose-response curves for the treatment of HMLER-shEcad cells with **1** after 72 incubation in the presence and absence of PGE2 (20 µM).

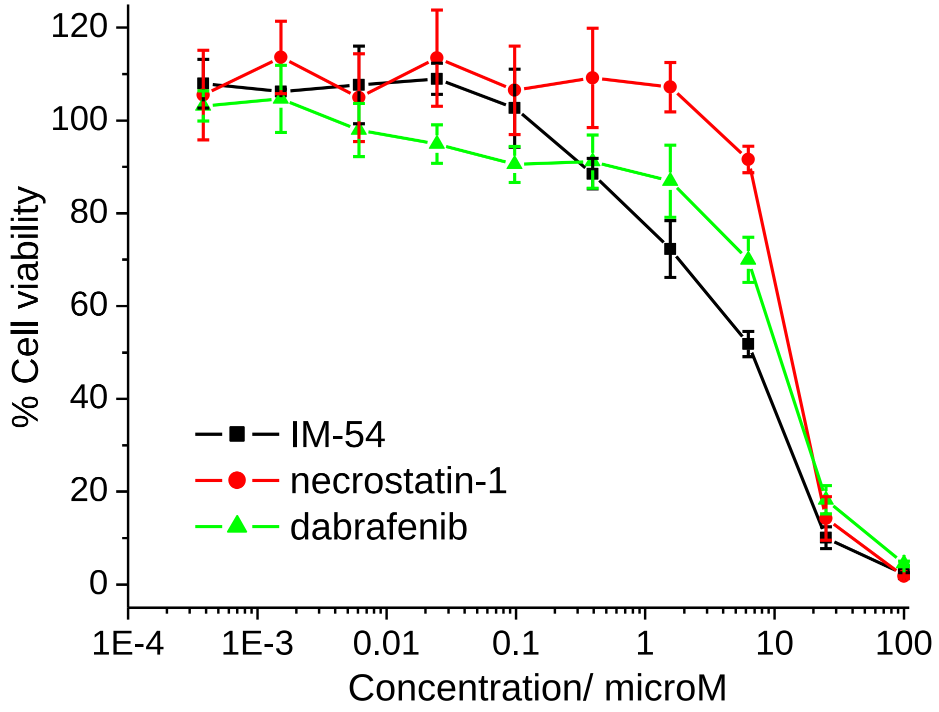

Figure S22. Representative dose-response curves for the treatment of HMLER-shEcad cells with **1** after 72 incubation in the presence and absence of IM-54 (10 μM), necrostatin-1 (20 μM), or dabrafenib (20 μM).

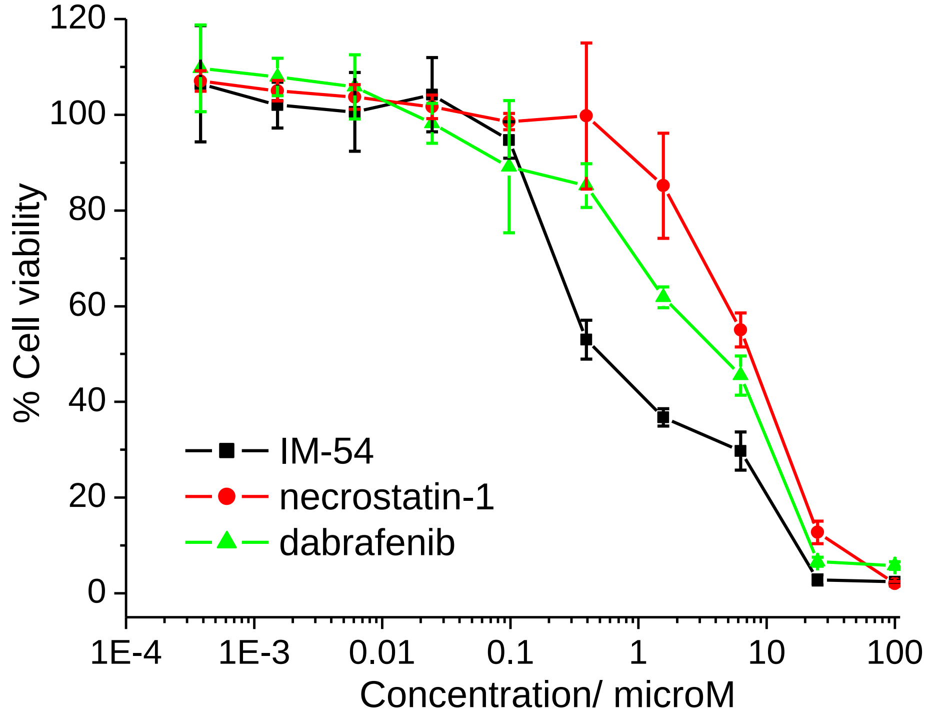

Figure S23. Representative dose-response curves for the treatment of HMLER-shEcad cells with **3** after 72 incubation in the presence and absence of IM-54 (10 μM), necrostatin-1 (20 μM), or dabrafenib (20 μM).

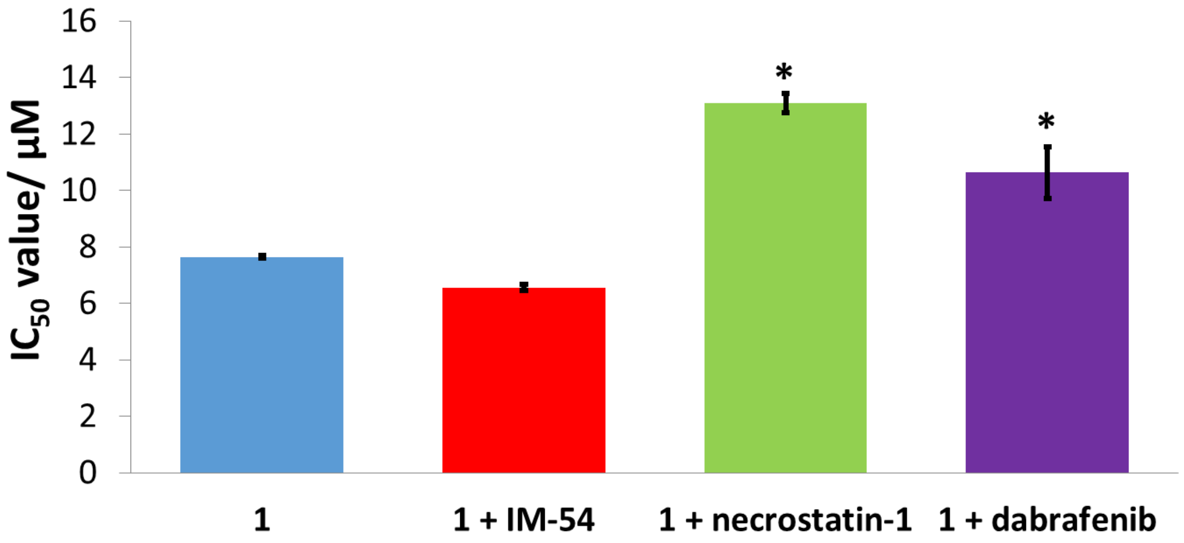

Figure S24. Graphical representation of the IC_{50} values of **1** against HMLER-shEcad cells in the absence and presence of IM-54 (10 µM), necrostatin-1 (20 µM), or dabrafenib (10 µM). Error bars represent standard deviations and Student t-test, * = p < 0.05.
